# Application of a biotin functionalized QD assay for determining available binding sites on electrospun nanofiber membrane

**DOI:** 10.1186/1477-3155-9-48

**Published:** 2011-10-24

**Authors:** Patrick Marek, Kris Senecal, Dawn Nida, Joshua Magnone, Andre Senecal

**Affiliations:** 1 Food Safety and Defense Team, U. S. Army Natick Soldier Research, Development and Engineering Center, 15 Kansas St. Natick M. A. 01760-5018, USA; 2 Molecular Sciences and Engineering Team, U. S. Army Natick Soldier Research, Development and Engineering Center, 15 Kansas St. Natick M. A. 01760-5018, USA

## Abstract

**Background:**

The quantification of surface groups attached to non-woven fibers is an important step in developing nanofiber biosensing detection technologies. A method utilizing biotin functionalized quantum dots (QDs) 655 for quantitative analysis of available biotin binding sites within avidin immobilized on electrospun nanofiber membranes was developed.

**Results:**

A method for quantifying nanofiber bound avidin using biotin functionalized QDs is presented. Avidin was covalently bound to electrospun fibrous polyvinyl chloride (PVC 1.8% COOH w/w containing 10% w/w carbon black) membranes using primary amine reactive EDC-Sulfo NHS linkage chemistry. After a 12 h exposure of the avidin coated membranes to the biotin-QD complex, fluorescence intensity was measured and the total amount of attached QDs was determined from a standard curve of QD in solution (total fluorescence vs. femtomole of QD 655). Additionally, fluorescence confocal microscopy verified the labeling of avidin coated nanofibers with QDs. The developed method was tested against 2.4, 5.2, 7.3 and 13.7 mg spray weights of electrospun nanofiber mats. Of the spray weight samples tested, maximum fluorescence was measured for a weight of 7.3 mg, not at the highest weight of 13.7 mg. The data of total fluorescence from QDs bound to immobilized avidin on increasing weights of nanofiber membrane was best fit with a second order polynomial equation (R^2 ^= .9973) while the standard curve of total fluorescence vs. femtomole QDs in solution had a linear response (R^2 ^= .999).

**Conclusion:**

A QD assay was developed in this study that provides a direct method for quantifying ligand attachment sites of avidin covalently bound to surfaces. The strong fluorescence signal that is a fundamental characteristic of QDs allows for the measurement of small changes in the amount of these particles in solution or attached to surfaces.

## Background

Non-woven fiber materials comprised of nano-scale electrospun fibers have unique properties and are being developed for use in filter media, scaffolds for tissue engineering, protective clothing, reinforcement in composite materials and sensors [1]. Nanofiber materials have a large surface area per unit mass on the order of 10^3 ^m^2^/g [2] and can easily be functionalized [1]. Nanofiber materials can be produced by an electrospinning process, during which nanofibers are created from an electrically charged jet of polymer solutions or polymer melts [1,3,4]. Nanofibers produced by electrospinning normally result in a fiber laden, nonwoven mat or membrane of randomized fiber orientation, size and spatial separations (pores). The origin of the randomness for which the electrospun nanofiber mat is known has been described as a chaotic oscillation of the spinning jet [5] and as a jet whipping and bending instability at the nozzle tip [6]. Research has been conducted on using electrospun membranes as sensors and as substrates for immunoassays [7-12]. Recently, electrospun nanofiber membranes have been demonstrated as a promising technology for biological agent capture and detection [12]. In biosensor applications, it is important to functionalize the fibers with ligands and chemistries in a consistent and repeatable manner so that detection and quantitation of analytes is reproducible. The density of binding sites is an important characteristic for sensor development [13]. Because of the complexity of non-woven electrospun membranes it would be of value to determine the optimum physical characteristics (as determined by weight during production) that provides the greatest number of available antibody attachment sites for assay development. Increasing the quantity of nanofibers per square cm will increase the surface area of the mat and the potential number of binding sites for antibody attachment. However, additional fibers are added only to the z-plane, increasing the thickness of the membrane, and potentially subjecting the signal of fluorescence based assays to attenuation.

**Figure 1:**
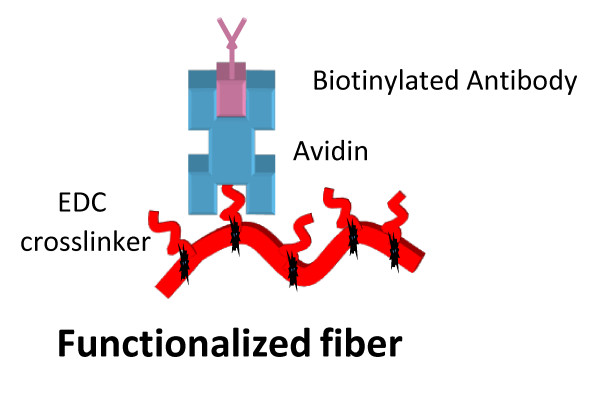
**Functionalized nanofiber**. Shows method used for attaching antibodies to PVC-COOH nanofibers.

Previously it was demonstrated that PVC-COOH nanofiber material could be functionalized with antibodies using a directional orientated two step: avidin protein - biotinylated antibody linkage [12] (Figure 1). Methods identified to determine the amount of avidin protein covalently attached to the membrane for assay development were found to be inadequate. Commonly used methods such as Micro BCA and Modified Lowry (Thermo Fisher Scientific, Rockford IL) are inefficient to quantify protein covalently attached to the nanofibrous membrane. These assays are designed to verify protein concentrations in solution and not proteins bound to a surface. Additionally, they lack the desired sensitivity for samples with protein concentrations in the nanogram range (2 - 40 ug/ml Micro BCA and 10 - 1500 ug/ml Modified Lowry). Alternative methods to quantify proteins absorbed to a surface via Nano Orange (Invitrogen), amido black staining and quartz crystal microbalance (QCM) showed assay sensitivities into the nanogram per cm^2 ^[14]. The Nano Orange fluorometric analysis is the only method to give absolute quantities of surface-bound proteins measure after the adsorbed protein was liberated from the surface using sequential rinse cycles of undiluted ethanol and distilled deionized water, concentrated with solvent evaporated [14].

The purpose of this study was to develop a new method for determining the amount of avidin protein covalently attached to complex nonwoven surfaces. Here we describe a fluorescence based method using QDs, taking advantage of their high quantum yield and excellent photostability, to quantify avidin immobilized by covalent attachment on nanofiber material with a direct measurement.

## Results

### Inhibition of membrane autofluorescence

**Figure 2:**
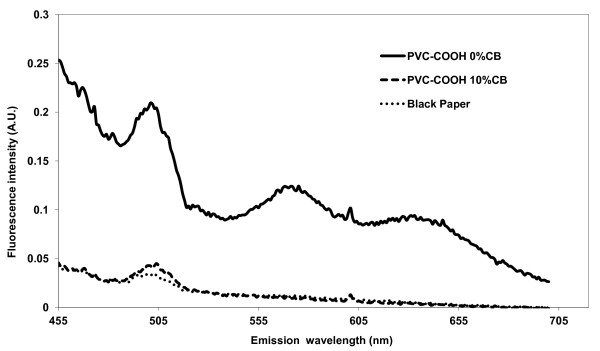
**Emission spectra of PVC-COOH membrane**. Fluorescence emission spectra of PVC-COOH electrospun nanofiber membrane with and without 10% w/w carbon black at 400 nm excitation. Fluorescence was measured on an Aminco Bowman II front face spectrophotometer.

PVC-COOH membranes demonstrated a broad autofluorescence signature upon excitation with various wavelengths from 280 nm to 400 nm. Figure 2 shows that when excited at 400 nm the electrospun membrane revealed significant autofluorescence emission between 455 nm and 705 nm. Although the intensity decreased towards the red region, the emission of a red emitting fluorescent reporter would still be interfered with by the background signal. Adding a 10% w/w carbon black powder to the polymer prior to solubilization in DMF and electrospinning significantly reduced the fiber autofluorescence, especially in the red region, Figure 2. The autofluorescence emission spectrum of PVC-COOH containing 10% CB decreased steadily (nearly linear) moving towards longer wavelength visible light (450 to 700 nm) while the untreated PVC-COOH had multiple emission peeks. Emission intensities of the CB containing nanofibers in the 635 to 700 nm wavelength range were nearly zero making it an ideal wavelength range for a direct measure assay.

### QD surface assay

**Figure 3:**
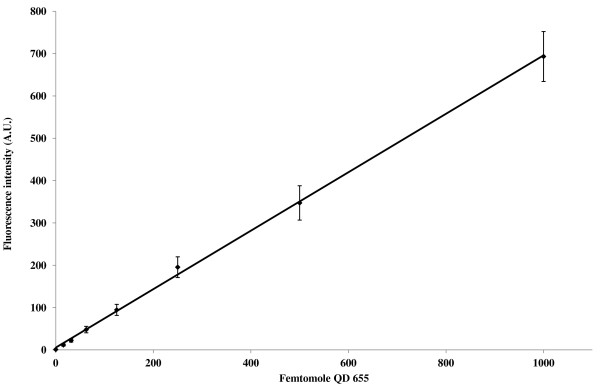
**Standard curve: femtomoles QD vs. fluorescence**. The fluorescence intensity was measured from QD 655 nm standard solutions (1000, 500, 250, 125, 62.5, 31.5, 15.6 and 0 femtomole in TBS) in a black 96 well plate (n = 3, 100 ul). Linear regression: y = 0.6907x + 5.3062 (R^2 ^= 0.999).

**Figure 4:**
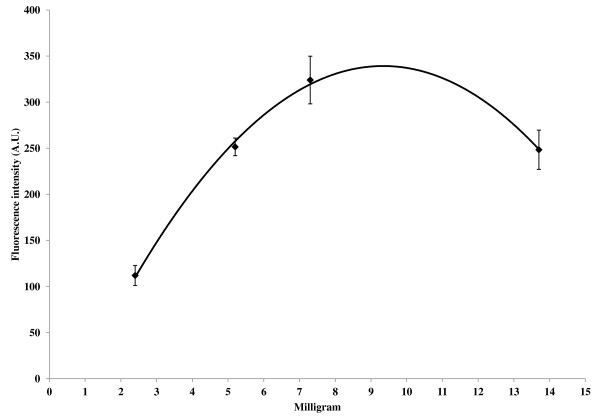
**Relationship of fluorescence and membrane weight**. Electrospun membrane material (PVC-COOH 10%w/w CB) of differing weights functionalized with avidin was interrogated with biotin-QD complexes. The fluorescence intensity (mean ± standard error, n = 18) has a 2^nd ^order polynomial relationship to the membrane weight, y = -4.757x^2 ^+ 88.878x - 75.945 (R^2^= 0.9973).

A standard curve for the concentration of QD 655 in solution versus fluorescent intensity produced a linear response (R^2 ^= .999), Figure 3. The relationship between fluorescence and QD concentration was used to calculate the amount of avaliable biotin (ligand) binding sites of avidin bound to the nanofiber membrane by the QD 655 binding assay. The relationship of total fluorescence to binding site numbers was hypothesized to have a linear response, similar to what is seen in the standard curve. The QD binding assay was used to measure the total fluorescent response in relation to the covalent binding of avidin to different weights of nanofiber mats (Figure 4). Results showed a nonlinear response to increasing fiber mat weights. The relationship of the total fluorescence to membrane spray weight appears to be represented accurately with a second order polynomial equation (R^2 ^= .9973) within the boundaries of the data set. The initial increase in fluorescence from 2.4 to 7.3 does have linear trend but as the weight of the nanofiber mat increases so does the thickness and congestion of the fibers resulting in a decrease in signal for the heaviest fiber mat tested. Fluorescence intensity reached a maximum for the 7.3 mg fiber mat sample. Table 1 summarizes the data for the femtomoles of QD bound to avidin on the surface for the different weights of nanofiber mats as determined by the equation (fluorescence intensity- 5.3062) ÷ 0.6907 = femtomoles of QD 655). Using only the linear portion of the fluorescence values from figure 4 it can be determined that on average 65 femtomole of QD can bind to 1 mg of nanofiber material. It can then be extrapolated that the heaviest sample at 13.7 mg should theoretically bind 890.5 femtomoles QD, 2.5x the amount calculated using the relative fluorescent values measured from the sample.

**Table 1:** Calculated femtomoles of QD 655 nm bound to avidin on the surface of electrospun nanofibers

Membrane	Average	SEM	n	Femtomole
Weight	Fluorescence			Qdot 655 nm
2.4	111.93	10.86	18	154.38
5.2	251.48*	9.56	18	356.43
7.3	324.00	25.85	18	461.43
13.7	248.35*	21.33	18	351.89

*No significant difference (p-value > 0.05).

## Discussion

Previously we were successful in developing an electrospun membrane sensor by covalently attaching avidin to the surface of the nanofibers for functionalization with biotinylated antibodies [12]. However, because the proteins are chemically attached to the surface and not in solution, we have been unable to quantify the antibody receptor sites on the nanofiber membrane mats. We attempted to use conventional protein assays (Modified Lowry and Micro BCA) to quantify the amount of surface bound avidin in order to determine the amount of ligand binding sites. We were unable to measure the amount of avidin protein attached to the nanofiber material with these assays, either because the amount of protein was below the assays sensitivity limits or because these assays are designed to quantify proteins in solution and not protein bound to a surface. We determined that an assay needed to be developed that was sensitive in the nanogram range to quantitate protein bound to a surface. The avidin protein can be attached to a surface and still maintain the biotin binding functionality allowing attachment of biotin labeled receptors. The strong binding affinity of avidin for biotin (Ka = 10^15 ^M^-1^) has made it useful for bioanalytical applications and immobilization of proteins to surfaces [13,15]. Therefore, we designed an assay that utilizes attachment other molecules to biotin, while still maintaining the strong affinity of biotin to avidin. We chose QDs attached to biotin as our reporter molecules for the assay.

The inherent properties of QDs make them useful tools for quantitation assays. They have been identified to have optical advantages in fluorescence detection when compared to conventional organic fluorophores [16]. QDs advantages over traditional organic dyes include the brightness originating from the high extinction coefficient, large Stokes shift and photostability, while having a comparable quantum yield to traditional organic fluorescent dyes [17]. It has been estimated that quantum dots are 20 times brighter and 100 times more stable than traditional fluorescent reporters [18]. The photostability and brightness of QDs make them ideal labels for developing an assay to measure surface bound moetities since multiple readings and long exposures to excitation light may be necessary to achieve sensitivities in femtomole range including sensitive photomultiplier tube (PMT) based systems. Materials such as PVC, used to produce electrospun nanofiber membranes in this study, can have an autofluorescence signature. Depending on the spectral response of the material at a specific excitation it may be difficult to find a fluorophore that is not hindered by the material autofluorescence intensity and profile. We found incorporating carbon black into the PVC-COOH spin dope almost completely diminished nanofiber autofluorescence. Utilizing both carbon black and QDs we were able to achieve sensitivity and very low sample noise.

**Figure 5:**
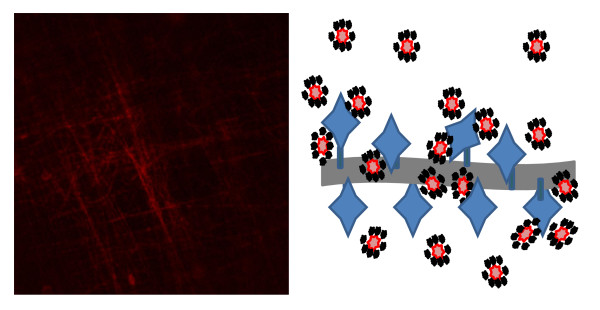
**Image of Qdot labeled nanofibers and binding reaction**. The fluorescent CLSM image (left) taken shows the uniform attachment of Qdot 655 nm to bound avidin on PVC-COOH nanofibers containing 10%w/w carbon black. The graphical representation (right) show the binding scheme used in the quantification of available biotin binding sites: where avidin is covalently attached to PVC-COOH nanofiber via carbodiimide chemistries (EDC and Sulfo-NHS) and biotin-Qdot complexes bind preferentially to avidin.

In this study, non avidin functionalized PVC-COOH membranes were exposed to high concentrations of the biotin-QD 655 complex to determine the extent of nonspecific binding to the nanofiber material. Previously we had determined that protein like immunoglobulins will nonspecifically absorb to the surface of nanofibers and are not easily washed off (unpublished data). However in this study it was found that the biotin-QD complex did not readily stick to the fibers and only accounted for a fluorescence signal intensity < 2.0 after washing. Here we demonstrated a QD 655 binding assay as a technique to measure the overall fluorescence response for avidin covalently attached onto nanofiber mats. Fluorescent confocal microscopy verified the labeling of covalently attached avidin to electrospun nanofibers with QDs, Figure 5. Two assumptions were made; first only one biotin functionalized QD would be able to bind to a single biotin binding site located on avidin, and second the fluorescent intensity of QDs in solution would be comparable to QDs attached to a surface. The second assumption is based on the understanding that, QDs are not known to quench in close proximity [19]. Consequently, by measuring the fluorescence intensity of bound biotin-QD complex, one should be able to calculate the moles of available binding sites from the covalent attached avidin protein located on the surface of nanofibers. Once the method was developed, it was utilized it to determine the relationship between available antibody binding sites to different weights of electrospun nanofiber mats. Increasing fiber mat weight results in fiber mats of similar diameter having a greater total number of fibers thereby increasing the carboxylated functional groups available for avidin attachment. The experimental results (Figure 4) showed that the different membrane weights did not have a linear response. Fluorescence intensity for the different membrane weights reached a maximum at 7.3 mg then fell sharply at 13.7 mg. It is hypothesized that increasing the fiber mass beyond 7.3 mg causes the anterior fibers to attenuate both excitation and emission of the QDs located on posterior nanofibers. The polynomial relationship described here may purely be an artifact generated from line of site when using fluorescence labeled reporters combined with complex nanofiber mats. It is anticipated that measurement of binding sites on a planar system using this method would maintain a linear response then plateau at a saturation point. However, in this study the added electrospun fibers are done so in a vertical manner (in the z-plane), affecting the linearity of the relationship for fiber weight vs. fluorescence intensity due to signal attenuation. The strong fluorescence signal that is a fundamental aspect of quantum dots has the promise to allow for measurement of small changes in the amount of these particles in solution or attached to a planar surface. More investigation is still needed to determine the limits for utilization of QDs for quantitation on more complex structures like the nanofiber mats investigated here.

## Conclusions

A QD assay was developed that allowed for the determination of available ligand binding sites of avidin chemically attached to surfaces. The assay lost its linearity when the thickness of the electrospun nanofiber mat was increased above a threshold. It is hypothesized that a shadowing effect (line of site) maybe taking place where the anterior fibers were blocking quantum dots located on posterior nanofibers. The crowding of fibers has the potential to block excitation and emission of bound QDs. It is anticipated that measurement of binding sites on a planar system using this method would maintain a linear response before a saturation point then plateau. The strong fluorescence signal that is a fundamental aspect of quantum dots has the promise to allow for measurement of small changes in the amount of these particles in solution or attached to a surface. Data observed could help with optimizing electrospun nanofiber membrane design for sensor development. More investigation is still needed to determine the limits for utilization of QDs for quantitation on more complex structures like the nanofiber mats investigated here.

## Materials and Methods

### Electrospinning device

The electrospinning apparatus used consisted of a DC power source (Gamma High Voltage Research, Inc. Model ES 30P-5W/DAM) where the charged positive electrode wire was coupled to a blunt end 22 gauge syringe containing polymeric solution. The polymer solution was drawn into the disposable 5 ml syringe (polypropylene) and mounted into KD scientific syringe pump model 780100 and flow rate set to 0.02 ml/h. An 18 AWG ground wire from the power source was attached to a conducting copper plate holding a 6.4 cm diameter screen, consisting of 100 mesh, woven 0.0045 inch T304 stainless steel wire. A voltage of 14 Kv was applied to the syringe with a gap distance of 17.5 cm from the collector.

### Polymer solutions for electrospinning

The polymer used to fabricate electrospun membranes was polyvinyl chloride 1.8% carboxylated (PVC-COOH) (Aldrich Chemical, St. Louis, MO). This polymer was solublized at 10% by weight in 80% dimethyl formamide (DMF) and 10% tetrahydrofuran (THF) w/w mixed with a magnetic stirplate for 24 h at room temperature. PVC-COOH nanofiber polymer material itself has a broad autofluorescence signature and emission scans at various wavelengths within the 450 nm to 800 nm range. Carbon black (CB) was added to the spin dope (10% weight of polymer), to lessen the autofluorescence of the polymer, then sonicated over night and mixed constantly on a magnetic stirrer until the polymer was electrospun.

### Fiber weight of electrospun membranes

Different weights of fiber mats were produced for assay development. Milligram quantities of fiber were electrospun on 6.4 cm diameter stainless steel screens. Screen weights were taken before and after electrospinning to determine total weight of fibers deposited on the screens. Fiber mats at total weights of approximately 2.4, 5.2, 7.3 and 13.7 mg were used in the study. From each 6.4 cm fiber mat, 18 smaller 0.75 cm circles were produced using a die cutter for the QD assay development in 96 well plates. Further mention of fiber weights in this paper will refer to the weights of the fibers produced by electrospinning on the 6.4 cm stainless steel screens, since fiber weights of the small 0.75 cm screen punches could not be measured with accuracy.

### Avidin attachment to electrospun membranes

Avidin was covalently attached to the carboxylated PVC using 1-ethyl-3-(3-Dimethylaminopropyl) carbodiimide Hydrochloride (EDC) in the presences of N-Hydroxysulfo-succinimide (Sulfo-NHS) (Thermo Fisher Scientific, Rockford, IL) with some modification [7]. Each of the 0.75 cm fibers were placed in individual wells of a 24 well tissue culture plate and wetted with 1 ml phosphate bufferend saline (PBS)/Tween 20 0.3% pH 7.2, soaked for 5 min and then rinsed with 500 ul of pH 5.0, 0.1 M 2-[N-morpholino] ethane sulfonic acid (MES)/0.1% Tween 20, 5 min shaking at 75 rpm on an orbital shaker. The wash solution was removed and 500 ul of fresh MES/0.1% Tween was added. Carboxyl groups on the nanofiber membranes were activated with 100 ul of EDC (10 mg/ml in MES Tween 0.1% pH 5.0) and 100 ul of Sulfo-NHS (27.5 mg/ml in MES Tween 0.1% pH 5.0) added to each well, shaken for 5 min at 75 rpm and then incubated for 30 min statically. Membranes were then washed twice in 1 ml of PBS (100 mM sodium phosphate, 150 mM NaCl, pH 7.2) to remove un-reacted EDC and Sulfo-NHS before a final 500 ul volume of avidin-PBS solution (200 ug/ml, PBS pH 7.4) was added to each membrane and shaken at 75 rpm for 1 h then static incubation overnight at 4°C.

### Attachment of QD

Each avidin coated membrane was washed 3 times in a Tris-buffered saline (TBS) containing 0.05% Tween 20 pH 8.0 on an orbital shaker for 5 min at 75 rpm. The final wash solution was removed before addition of the biotinylated QDs. Biotinylated QD (QDot 655, Invitrogen Corp. Carlsbad CA.) was prepared in TBS pH 8.0 at a concentration of 5 nM and 500 ul was added to each well for static incubation, 1 h at RT and then overnight at 4°C. Following overnight incubation each membrane was washed 3 times in TBS pH 8.0 containing 0.05% Tween 20 at 75 rpm for 5 min.

### Measurement and analysis

Each QD 655 coated membrane was transferred to a black 96 well micro titer plate being careful to orientate the electrospun nanofibers facing up. Each membrane was covered with 100 ul of TBS pH 8.0 to prevent dehydration and quenching of the QDs during measurement of fluorescence. A standard curve was generated (total fluorescence vs. femtomole of QD 655) from a serial dilution series of the stock 2 uM QD 655 solution at 1000, 500, 250, 125, 62.5, 31.5, 15.6 and 0 femtomoles of QDs contained in100 ul of TBS pH 8.0,measured in triplicate. The samples were read on a fluorescence plate reader (Fluoroskan, Thermo Fisher Scientific) using a normal beam size and an integration time of 1000 ms (320 nm excitation and 650 nm emission filter set). Control membranes for measurement of fluorescence background and nonspecific binding of the QDs to the nanofibers were also included in the assay. The nonspecific binding was measured on membranes that received a dose of biotinylated QD 655 but were not activated with EDC and Sulfo-NHS.

### Confocal laser scanning microscopy (CLSM)

Images of QD labeled electrospun nanofibers were taken on a Carl Zeiss LSM 710 (Thornwood, NY) confocal microscope using an EC Plan-Neofluar Iris M27, 100x objective (NA 1.3, oil). The sample was excited using the 405 nm diode laser (30%, 1.0 × zoom, pinhole 66 um) and the emission detection was set from 635 nm to 678 nm capturing the narrow emission peek of the 655 quantum dot.

### Statistical Analysis

Statistical analysis was performed using Statistical Analysis Systems software version 9.1 (SAS Institute, Inc., Cary, N.C.). Regression analysis was conducted to determine the line of best fit for both the standard curve and membrane weight data sets. The means of each concentration or treatment level for both the standard curve (n = 3) and the membrane weight data (n = 18) were used for linear and 2^nd ^order polynomial analysis using the REG procedure respectively. Analysis of variance was conducted using the MIXED model procedure for the membrane weight data with significant differences (P ≤ 0.05) between LSMEANS determined by the PDIFF statement.

## Competing interests

The authors declare that they have no competing interests.

## Authors' contributions

KS and AS conceived of the study and contributed to data interpretation. PM and JM performed QD binding assays and PM also conducted statistical analysis and LSCM imaging. DN performed electrospinning with KS. PM, KS and DN all contributed to writing the manuscript. AS and JM reviewed and revised the manuscript. All authors have read and approved the final manuscript.
